# Occurrence of apomictic conspecifics and ecological preferences rather than colonization history govern the geographic distribution of sexual *Potentilla puberula*

**DOI:** 10.1002/ece3.6455

**Published:** 2020-06-22

**Authors:** Flavia Domizia Nardi, Karl Hülber, Dietmar Moser, Henar Alonso‐Marcos, Andreas Tribsch, Christoph Dobeš

**Affiliations:** ^1^ Department of Forest Genetics Austrian Research Centre for Forests Vienna Austria; ^2^ Department of Biosciences University of Salzburg Salzburg Austria; ^3^ Department of Conservation Biology, Vegetation Ecology and Landscape Ecology University of Vienna Vienna Austria

**Keywords:** apomixis, ecological preferences, European Alps, geographic distribution, postglacial colonization, reproductive interference

## Abstract

The geographic distribution of sexual‐apomictic taxa (i.e., comprising individuals usually reproducing either sexually or asexually via seeds) is traditionally thought to be driven by their ecological preferences and colonization histories. Where sexuals and apomicts get into contact with each other, competitive and reproductive interactions can interfere with these factors, an aspect which hitherto received little attention in biogeographic studies. We disentangled and quantified the relative effects of the three factors on the distribution of tetraploid sexuals in *Potentilla puberula* in a latitudinal transect through the Eastern European Alps, in which they are codistributed with penta‐, hepta‐, and octoploid apomictic conspecifics. Effects were explored by means of binomial generalized linear regression models combining a single with a multiple predictor approach. Postglacial colonization history was inferred from population genetic variation (AFLPs and cpDNA) and quantified using a cost distance metric. The study was based on 235 populations, which were purely sexual, purely apomictic, or of mixed reproductive mode. The occurrence of apomicts explained most of the variation in the distribution of sexuals (31%). Specifically, the presence of sexual tetraploids was negatively related to the presence of each of the three apomictic cytotypes. Effects of ecological preferences were substantial too (7% and 12% of the total variation explained by ecological preferences alone, or jointly with apomicts’ occurrence, respectively). In contrast, colonization history had negligible effects on the occurrence of sexuals. Taken together, our results highlight the potentially high impact of reproductive interactions on the geographic distribution of sexual and apomictic conspecifics and that resultant mutual exclusion interrelates to ecological differentiation, a situation potentially promoting their local coexistence.

## Graphical abstract

Geographic distributions of taxa are the result of several interrelated factors. In our study, we quantified and separated the role of three main drivers (ecological preferences, colonization history, and competitive and reproductive interactions with conspecifics) on the distribution of sexual *Pontentilla puberula* (Rosaceae) in the Eastern European Alps. The occurrence of apomictic individuals was the main factor affecting sexuals' distribution, followed by ecological preferences and their joint effects. Our results highlight the interplay of competitive exclusion by conspecifics and ecological differentiation in determining mosaic‐like geographic distributional patterns.

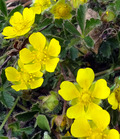

## 1 INTRODUCTION

Given their complex nature, geographic distributions of organisms are determined by several interrelated factors (e.g., Sheth, Morueta‐Holme, & Angert, 2020). The most evident one is the dependence on specific environmental conditions, commonly used to predict species distributions (e.g., Austin, 2007; Elith & Leathwick, 2009; Franklin, 2013; Guisan & Thuiller, 2005). However, the actual geographic range of a species (i.e., its realized niche) is usually smaller than the ecologically suitable area (i.e., its potential niche; Jackson & Overpeck, 2000). This implies that other factors codetermine species distributions (Gaston, 2003; Hutchinson, 1957; Peterson & Soberón, 2012; Sexton, McIntyre, Angert, & Rice, 2009; Soberon & Arroyo‐Peña, 2017).

Limited propagule dispersal abilities (i.e., the presence of geographic barriers) might cause ecologically suitable areas to be unoccupied (Peterson & Soberón, 2012). Such effects of the colonization history are expected to be particularly strong in regions characterized by severe climatic fluctuations, like Pleistocene glacial‐interglacial cycles. In these areas, the current geographic distribution of species may depend more strongly on the degree of dispersal limitation during the postglacial recolonization of formerly glaciated areas than on current ecological conditions, resulting in “incomplete range filling” (Kissling, Blach‐Overgaard, Zwaan, & Wagner, 2016; Svenning & Skov, 2007).

Interactions with co‐occurring species (Callaway & Walker, 1997), a third factor potentially affecting species distributions, have received less attention beyond the local scale (reviewed by Wisz et al., 2013). Thereby, the influence of closely related taxa can be presumed to be remarkably strong due to additional effects of reproductive interactions (Bullock, Edwards, Carey, & Rose, 2000; Hardin, 1960; Violle, Nemergut, Pu, & Jiang, 2011; Wisz et al., 2013). For instance, closely related species or intraspecific ploidy cytotypes may tend to exclude each other due to the combined effects of resource competition and reproductive interference, that is, loss of female gametes by interspecific or inter‐cytotype crosses (Hülber et al., 2015; Kyogoku, 2015; Levin, 1975).

Reproductive interactions among co‐occurring cytotypes are of particular interest in plant species comprising sexual and apomictic individuals (i.e., reproducing asexually via seeds; Asker & Jerling, 1992). Most of such sexual‐apomictic systems are characterized by an association between ploidy level and reproductive mode, with lower ploidy levels reproducing usually by outcrossing sexuality and higher ploidy levels mostly apomictically, respectively (Asker & Jerling, 1992; Hörandl, 2010). Interactions between reproductive modes are likely to be asymmetric (Dobeš et al., 2018; Hersh, Grimm, & Whitton, 2016). Apomictic individuals commonly produce meiotically reduced pollen capable of fertilizing sexual individuals (van Dijk, 2003; Mogie, Britton, & Stewart‐Cox, 2007). Conversely, the embryos of apomicts develop parthenogenetically, precluding fertilization of the egg cell by pollen from sexuals. Thus, negative effects of cross‐fertilization such as reduced seed set (Dobeš et al., 2018; Hersh et al., 2016; Tas & Van Dijk, 1999) or reduced offspring fitness (Van Dijk, Tas, Falque, & Bakx‐Schotman, 1999) in apomicts can only be caused by developmental failures resulting from cross‐fertilization of the endosperm and can, hence, be expected to be lower than in sexuals. Moreover, cross‐fertilization might transmit the apomictic character (Grimanelli, Leblanc, Perotti, & Grossniklaus, 2001; Ozias‐Akins & van Dijk, 2007) into the population of sexuals contributing to their local replacement (Joshi & Moody, 1995, 1998; Mogie, 1992) and, consequently, limiting the distributional range of sexuals.

A major obstacle in understanding the distributions of taxa is the difficulty to disentangle the effects of the major factors, ecological preferences, colonization history, and occurrence of closely related taxa, which potentially interrelate in various ways: (a) spatial changes in environmental conditions frequently coincide with colonization pathways (i.e., historic effects), leaving ecological preferences and colonization abilities of cytotypes as plausible causes for the observed distribution patterns (Brown, Stevens, & Kaufman, 1996; Schinkel et al., 2016; Sexton et al., 2009). (b) Apomicts tend to be better colonizers than outcrossing sexuals due to factors related to apomixis itself, like uniparental reproduction and independence from pollinators (Baker, 1955; Kirchheimer et al., 2018; Stebbins, 1957), as well as traits related to polyploidy, such as bigger seeds and higher vigor (Te Beest et al., 2012). These advantages are substantial during the recolonization of a previously glaciated area (Bierzychudek, 1985; Hörandl, 2006). Moreover, residual sexuality and other sources of genetic variation (Hörandl & Paun, 2007) give facultative apomicts the opportunity to exploit different niches, thus increasing their colonization success. As a consequence, apomicts likely precede outcrossing sexuals during range expansions, further benefitting from inhibitory priority effects (Levins & Culver, 1971; Shulman et al., 1983), that is, blocking or delaying the range expansion of sexuals as a frequency‐dependent phenomenon (Britton & Mogie, 2001). Analogously, reproductive modes would reduce or even hinder each other's advance if their fronts meet during colonization from different refugia. (c) Finally, the joint action of ecological preferences of sexuals and apomicts and their reproductive interferences may either foster or hamper their coexistence. On the one hand, competition among closely related species or cytotypes may lead to niche segregation, mitigating negative effects of reproductive interference (Peers, Thornton, & Murray, 2013; Sonnleitner et al., 2016). On the other hand, apomicts may outcompete or reproductively suppress sexuals across their whole ecological niche, if their genetic diversity is kept high enough by frequent immigration of new clones or recurrent backcrossing with sexuals (Frozen Niche Variation model, Vrijenhoek, 1984; Vrijenhoek & Parker, 2009).

In the present study, we quantify the relative effects of ecological preferences, colonization history, and occurrence of conspecific apomicts on the distribution of sexual *Potentilla puberula* Krašan (Rosaceae) in the Eastern European Alps. We focus on sexuals because apomicts cannot be regarded as evolutionarily independent lineages, due to historic and rare contemporary unidirectional gene flow from sexuals to apomicts (Nardi et al., 2018). Several preconditions make this species a highly suitable model system: firstly, sexual and apomictic cytotypes occur in a sympatric mosaic‐like pattern despite their ecological differentiation (Alonso‐Marcos et al., 2019a). Secondly, a large part of the study area was covered by an ice sheet during the Last Glacial Maximum (LGM; Ehlers & Gibbard, 2004), making it an appropriate setting for a possibly differential colonization history of sexuals and apomicts. Finally, an asymmetric reproductive interference between reproductive modes has been found, with sexuals suffering a reduction in seed set after inter‐cytotype pollination (Dobeš et al., 2018). However, the relative effect of each factor on the distribution of sexual *P. puberula* is still unclear. To quantify their relative importance, we (a) reconstruct the postglacial recolonization pathway of sexuals into previously glaciated areas, using nuclear and plastid markers, to calculate a cost distance metric, and (b) combine this information with earlier acquired data on the ecological habitat preferences and occurrence patterns of reproductive modes at the population level in binomial generalized linear regression models based on both a single and a multiple predictor approach.

## 2 MATERIALS AND METHODS

### 2.1 Study species and area

*Potentilla puberula* Krašan (Rosaceae) is a herbaceous species inhabiting lowland to subalpine, moderately xeric grasslands and open dry forests in the European Alps and the Western Carpathians (Kurtto, Lampinen, & Junikka, 2004). It comprises five morphologically hardly differentiated ploidy cytotypes (Bigl, Paule, & Dobeš, 2019): tetra‐ (2*n* = 4*x* = 28), penta‐ (2*n* = 5*x* = 35), hexa‐ (2*n* = 6*x* = 42), hepta‐ (2*n* = 7*x* = 49), and octoploids (2*n* = 8*x* = 56). Tetraploids, whose distribution does not expand east of East Tyrol (Austria, Alonso‐Marcos et al., 2019a; Dobeš, 1999), reproduce nearly exclusively sexually and are self‐incompatible, whereas penta‐, hepta‐, and octoploids show a wider distribution (Alonso‐Marcos et al., 2019a; Dobeš, 1999) and reproduce predominantly via pseudogamous apomixis (i.e., embryos develop autonomously but fertilization of the polar nuclei or central cell is required for a regular development of the endosperm and the seed; Müntzing, 1928; Rutishauser, 1969) and are self‐compatible (Dobeš et al., 2013). The rate of sexuality was estimated by means of flow cytometric seed screening (Matzk, Meister, & Schubert, 2000) in these three cytotypes to be around 10% (Dobeš et al., 2013, 2018; Nardi et al., 2018). Hexaploids, which share the same distribution as the latter cytotypes (Dobeš, 1999), comprise both apomictic and sexual individuals, the latter originating from spontaneous autopolyploidization of tetraploids (Nardi et al., 2018), and were, hence, not considered in this study. A study based on amplified fragment length polymorphism (AFLP) markers revealed both an intraspecific origin of the apomictic genotypes and that the apomictic cytotypes do not represent distinct genetic lineages (Nardi et al., 2018). Rather, *P. puberula* with all its variation in ploidy and reproductive mode represents a single phylogenetic lineage and a complex evolving species.

**FIGURE 1:**
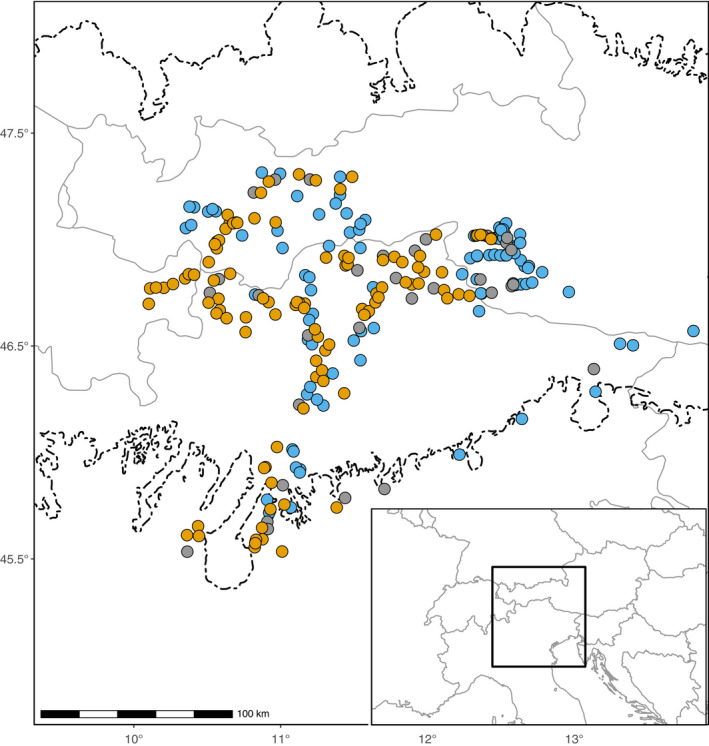
Cytotype composition of 235 populations of *Potentilla puberula* in a transect ranging from ice‐free areas beyond the southern margin of the ice sheet during the Last Glacial Maximum (dashed line) according to Ehlers et al. (2011) to the formerly (LGM) highly glaciated central parts of the Alps (up to the Inn valley). The inset shows the location of the transect on the Central European scale. Orange, blue, and gray circles represent sexual (tetraploid), apomictic (penta‐, hepta‐, and octoploid), and mixed sexual‐apomictic populations. Hexaploids (which can be either sexual or apomictic and therefore excluded from the analyses) are not shown. Redrawn in an adapted form from Alonso‐Marcos et al. (2019a, 2019b)

The study area is located within the European Alps (between 44.7° and 48.1°N latitude and 9.4° and 13.9°E longitude), ranging from the Garda lake area in the south to the Inn valley in the north and from the Lower Engadine in the west to the Julian Alps and Prealps in the east. We chose this area to cover all known occurrences of tetraploids in the Alps (Alonso‐Marcos et al., 2019a; Dobeš, 1999). Ploidy was screened on an average of 15 individuals from each of 235 populations by Alonso‐Marcos et al. (2019a), 137 of which comprised tetraploids (Figure 1, Table S1). Three populations studied by these authors were excluded from our investigation, due to either extreme geographic isolation (Oberegg, close to Salzburg, Austria) or presence of hexaploids only (Bagni Lusnizza and Roveredo, in Friuli, Italy). The sampling was designed to representatively cover the habitat conditions realized by the species within the study area.

### 2.2 Determination of the postglacial colonization pathway of sexuals

In order to reconstruct the postglacial colonization pathway of sexual tetraploid *P. puberula* into the study area, chloroplast sequence (cpDNA) and nuclear amplified fragment length polymorphism (AFLP) data were obtained for a subsample of 240 tetraploid individuals from 68 populations, published by Nardi et al. (2018). In brief, the molecular data consisted of a 1,108–1,177 bp combined sequence of the *trnH(gug)‐psbA* intergenic spacer (IGS) and the *rps16* intron, and of a presence–absence matrix of 370 AFLP loci (for details, see Nardi et al., 2018).

The cpDNA sequences were aligned using the program MAFFT (Katoh, 2002) with subsequent manual improvement on Geneious v.8.0 (http://www.geneious.com, Kearse et al., 2012). A region of 58 bp in the *psb*A‐*trn*H marker was removed because it included an inverted region, which showed homoplastic evolution with respect to the overall phylogeny of the marker (Whitlock, Hale, & Groff, 2010) and an only ambiguously alignable region linked to the latter. Indels were manually coded as single nucleotide polymorphisms (Simmons & Ochoterena, 2000).

For each population, four genetic indices were calculated: haplotype diversity (Nei & Tajima, 1981) and nucleotide diversity (Nei, 1987) of the cpDNA sequences using the R package pegas v.0.11 (Paradis, 2010); and gene diversity (Kosman, 2003; Nei, 1987) and rarity of AFLP markers (Schönswetter & Tribsch, 2005) using the R script AFLPdat v.2010 (Ehrich, 2006). In order to assess the population structure of tetraploid sexual *P. puberula*, the genetic differentiation among populations was estimated using *D*_est_ (Jost, 2008), and the presence of isolation by distance (Wright, 1943) was tested via a Mantel test based on the Pearson correlation coefficient with 10,000 permutations using the R package ade4 v. 1.7–13 (Dray & Dufour, 2007).

To infer the direction of the postglacial colonization of sexual *P. puberula* into the previously glaciated areas, we adapted a method applied in humans (Ramachandran et al., 2005) and *Arabidopsis thaliana* (François, Blum, Jakobsson, & Rosenberg, 2008; Lee et al., 2017). The study area was divided into 0.1° × 0.1° grid cells. For each cell, we correlated (by means of Spearman's rank correlation) the spatial distance from the cell's centroid to each population with the populations’ values separately for each genetic index. Assuming a single colonization source and no local introgression from other species at the populations (Nardi et al., 2018), both genetic diversity and rarity are expected to decrease from the colonization source along the colonization pathway due to genetic drift (Austerlitz, Jung‐Muller, Godelle, & Gouyon, 1997; Excoffier, Foll, & Petit, 2009; Nei, Maruyama, & Chakraborty, 1975; Schönswetter & Tribsch, 2005). Thus, cells at or close to the colonization source are indicated by strong negative correlation, while cells far from it are characterized by no or a positive correlation. For each genetic index, we then ranked the cells according to their correlation coefficients and finally combined the four rankings into a single one by averaging the ranks obtained for the single genetic indices. By correlating the combined ranks with the latitude of populations, it is possible to determine the general direction of colonization (north to south or south to north). Consequently, the colonization source inferred was defined as the respective margin (either the northern or southern one) of the LGM ice sheet (Ehlers, Gibbard, & Hughes, 2011).

### 2.3 Factors determining species distributions: ecological preferences, colonization history, and occurrence of apomicts

As ecological predictors, we used the variables acquired at each population by Alonso‐Marcos et al. (2019a) and available on Dryad (Alonso‐Marcos et al., 2019b): topographic data (elevation, inclination and aspect, i.e., slope exposure), bioclimatic data (bio04: temperature seasonality, bio12: annual precipitation, and bio15: precipitation seasonality; retrieved from the CHELSA climate database, Karger et al., 2017), a normalized vegetation differentiation index (NDVI, modis.gsfc.nasa.gov, Didan, 2015), and a land use indicator (oligohemerobic vs. mesohemerobic habitat). All continuously distributed variables were scaled to zero mean and unit variance prior to the analyses. Visual data exploration revealed an approximate normal distribution for all variables except for Bio12, which needed to be cubic‐root transformed. Aspect was measured as divergence from the north (0° in the north and increasing values toward east and west to 180° in the south).

To obtain an ecologically weighted measure for the distance that sexual tetraploid *P. puberula* had to cover during its migration from the margin of the LGM ice sheet to current populations, we used the least accumulative cost distance as implemented in the cost distance function of ESRI ArcGIS 10.5.0 (Environmental Systems Research Institute (ESRI), 2019). Since *P. puberula* is a predominantly lowland species and given the harsher climatic conditions at the end of the LGM, we assumed that colonization took place predominantly along the valley bottoms. Hence, elevation (100m Copernicus Land Monitoring EU‐DEM; https://www.eea.europa.eu/data‐and‐maps/data/copernicus‐land‐monitoring‐service‐eu‐dem) was used as a cost raster to define the impedance or cost to move planimetrically through each cell.

The presence/absences of penta‐, hepta‐, or octoploid individuals were used as binary predictor variables to assess the effect of the occurrence of apomicts on the distribution of sexual tetraploids.

### 2.4 Estimation of the effect of single predictor variables and the relative effects of the three main factors on sexuals’ distribution

We estimated the effects of ecological preferences, colonization history, and occurrence of apomicts on the occurrence (i.e., presence/absence) of sexual tetraploid *P. puberula* plants by means of binomial generalized linear regressions. Regressions were performed separately for each variable described in the previous section (i.e., single predictor approach). A Bonferroni correction was applied to *p* values to account for multiple comparisons. In order to determine the relative effect of the three main factors, we combined the variables representing ecological preferences (i.e., elevation, inclination, aspect, land use, NDVI, bio04, bio12, and bio15), occurrence of apomicts (presence/absence of the three apomictic cytotypes), and colonization history (cost distance) in a variation partitioning analysis (Borcard, Legendre, & Drapeau, 1992; i.e., a multiple predictor approach), following the logic of partial linear regressions as introduced by Legendre and Legendre (2012). This approach allows to determine and quantify the variation explained by each main factor and by a particular combination of two (e.g., by colonization history and ecological preferences) or even all three factors. This procedure extended to three explanatory matrices—as realized in the function varpart of the R library vegan v.2.5‐5 (Oksanen et al., 2018)—was adapted to binomial generalized linear regression models by using the adjusted *D* ^2^ (Guisan & Zimmermann, 2000) as a measure of the explained variation. *D* ^2^ represents the proportion of explained deviance weighted by the number of coefficients fitted in the model. It can be interpreted equivalently to the adjusted *R* ^2^ of linear regressions.

Additionally, we estimated the intrinsic effect of ploidy (as nucleotypic effects, Levin, 1983) on the occurrence patterns of apomictic cytotypes (penta‐, hepta‐, and octoploids). The occurrence of each cytotype was used as single binary predictor in binomial generalised linear regressions to explain the occurrence of the two other apomictic cytotypes in the 133 populations where apomicts were found.

If not mentioned otherwise, all the analyses were performed using R version 3.5.1 (R Development Core Team, 2018).

## 3 RESULTS

### 3.1 Direction of the postglacial colonization pathway of sexuals

The 68 genetically characterized tetraploid populations were weakly differentiated (*D*_est_ = 0.05) and isolation by distance was recognized (*R* ^2^ = .23, *p* < .001), compatibly with a single origin scenario.

**FIGURE 2:**
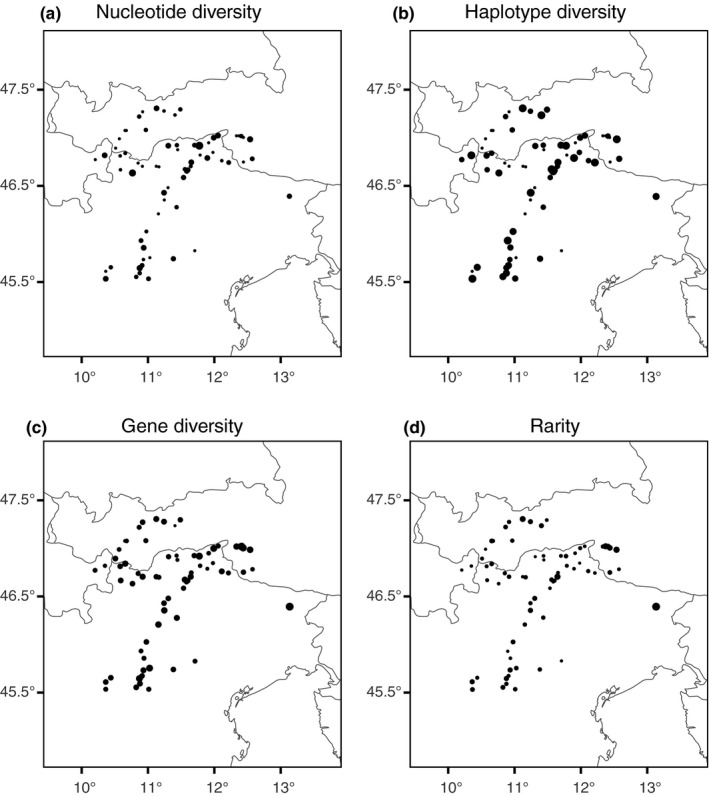
CpDNA‐ (a, b) and AFLP‐based (c, d) genetic indices of 68 tetraploid sexual populations of *Potentilla puberula* in the Eastern European Alps. Dot size is proportional to population values

**FIGURE 3:**
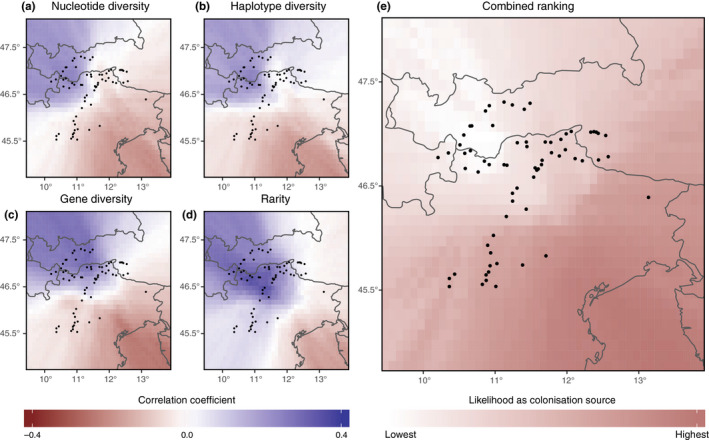
Probability of 0.1° × 0.1° cells of the study area to have served as Pleistocene refugium for tetraploid *Potentilla puberula*. The color of each cell illustrates the coefficient of a correlation between the spatial distance from a cell's centroid to each of 68 genetically characterized populations (black dots) and the populations’ values for a particular genetic index (a–d): negative and positive Spearman rank correlation coefficients are indicated in red and blue, respectively. Genetic indices of populations are based on cpDNA sequences (a, b) and AFLP fingerprints (c, d). A combined ranking of these four correlation coefficients for each cell is illustrated in (e): Areas that more likely have served as refugium at the LGM are represent in darker red, while those far from refugial areas are indicated in white

**FIGURE 4:**
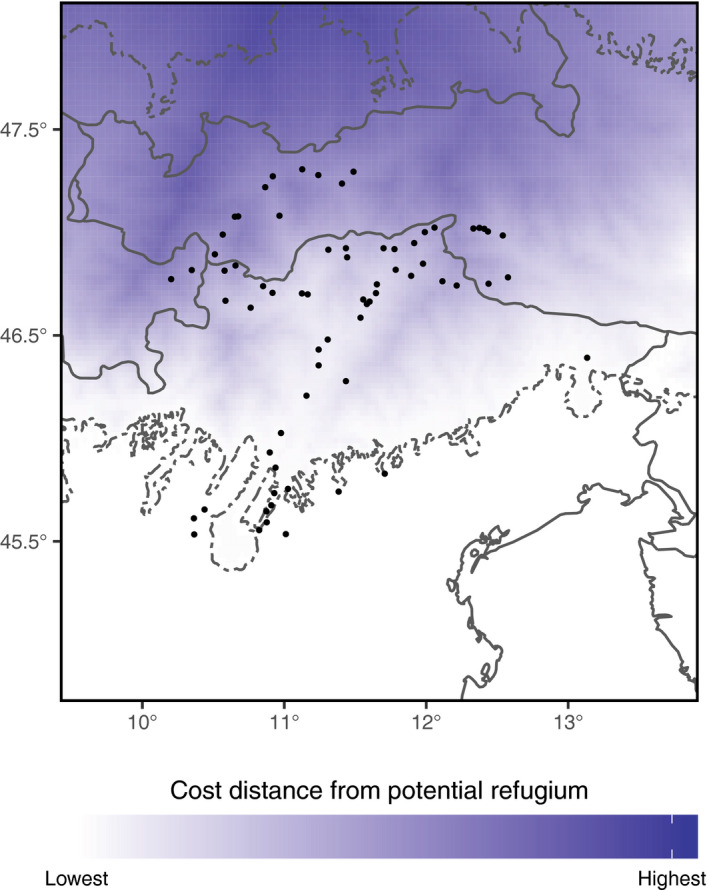
Cost distance surface of the study area assuming migration taking place predominately along the valley bottoms. Low and high cost distance values from the potential refugium at the southern border of the ice sheet at the LGM (dashed line; Ehlers et al., 2011) are given in white and blue, respectively. Black dots indicate the locations of 235 populations of *Potentilla puberula*

The four genetic indices (Table S1, Figure 2) showed a highly consistent pattern. The correlation coefficients (ranging from −0.22 to 0.24, from −0.21 to 0.25, from −0.26 to 0.29, and from −0.19 to 0.35 for the cpDNA‐derived nucleotide and haplotype diversities, as well as for the AFLP‐derived gene diversity and rarity, respectively) increased from southeast toward northwest (Figure 3a–d). Accordingly, the cells with the highest and lowest values of the combined rank were located in the southeastern and the northwestern part of the study region, respectively (Figure 3e). The combined ranks significantly correlated with latitude (*ρ* = 0.74, *p* < 0.001). We, thus, identified the southern LGM ice sheet margin as colonization source of the formerly glaciated area and calculated the cost distances accordingly. Cost distances to all populations are reported in Table S1, and the cost distance surface is shown in Figure 4.

### 3.2 The effect of ecological preferences, colonization history, and occurrence of apomicts

**FIGURE 5:**
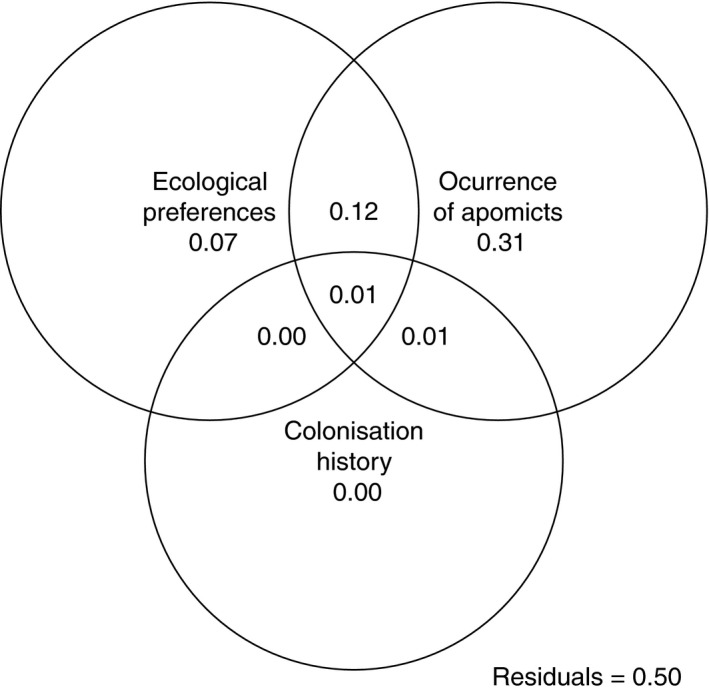
The importance of ecological preferences, colonization history, and the occurrence of higher‐ploid apomictic cytotypes for the occurrence of sexual, tetraploid *Potentilla puberula* in the Eastern European Alps. Values represent the proportion of variation explained by each of these factors or a combination of them. Residuals refer to the unexplained variation

The occurrence of apomicts was the most relevant factor determining the distribution of the sexual tetraploid cytotype. In the single predictor analysis, we found a significantly negative relationship between the presence of sexuals and that of apomicts within a population for each apomictic cytotype (see Table 1), being the effect of pentaploids by far the strongest (*D* ^2^ = 0.37). The multiple predictor analysis revealed that all apomictic cytotypes together explained nearly one third of the variation in the occurrence of sexuals (Figure 5). In addition, some variables representing ecological preferences were significantly related to the sexuals’ occurrence (Table 1): Steep slopes, low land use intensity, and low annual precipitation promoted the presence of sexuals. However, the common explanatory value of ecological preferences was much lower (7%, see Figure 5) compared to the occurrence of apomicts. An even higher proportion of variation (12%) was explained by both the effect of occurrence pattern and ecological preferences. In contrast, the effect of colonization history on sexuals’ distribution was negligible.

**TABLE 1:** Effects of variables representing ecological preferences, colonization history, and occurrence of apomictic cytotypes on the occurrence of sexual, tetraploid *Potentilla puberula* in the eastern European Alps derived from single predictor binomial generalized linear regressions

	Coefficients ± *SE*	*z* value	*p* values	*D* ^2^
Ecological preferences				
Elevation	−0.33 ± 0.14	−2.36	0.219	0.01
Inclination	0.58 ± 0.15	3.87	**0.001**	0.05
Aspect	0.12 ± 0.13	0.88	1.000	0.00
Land use	−2.24 ± 0.49	−4.54	**<0.001**	0.09
NDVI	0.06 ± 0.13	0.46	1.000	0.00
Bio04	−0.00 ± 0.13	−0.03	1.000	0.00
Bio12	−0.71 ± 0.16	−4.59	**<0.001**	0.07
Bio15	0.22 ± 0.13	1.64	1.000	0.00
Colonization history				
Cost distance	0.00 ± 0.00	−0.73	1.000	0.00
Occurrence of apomicts				
Pentaploids	−3.65 ± 0.41	−8.93	**<0.001**	0.39
Heptaploids	−1.56 ± 0.31	−5.11	**<0.001**	0.08
Octoploids	−2.49 ± 0.56	−4.47	**<0.001**	0.09

Note

*D* ^2^ is a coefficient of determination (equivalent to *R* ^2^) for generalized linear regression models following Guisan and Zimmermann (2000). *p* values significant after Bonferroni correction (adjusted *p* < 0.05) are given in bold. Bio04: temperature seasonality range (i.e., standard deviation of monthly mean temperatures × 100); bio12: annual precipitation; bio15: precipitation seasonality (i.e., coefficient of variation of monthly means of precipitation); cost distance: elevation weighted spatial distance to the potential Pleistocene refugium at the southern border of the ice shield at LGM; and penta‐, hepta‐, and octoploids: occurrence (i.e., presence/absence) of the respective cytotype. The negative regression coefficient for the categorical predictor land use indicates a higher probability of occurrence of tetraploids in (semi)natural rather than intensively used (more human shaped) sites, in comparison with sites inhabited by apomicts alone. Similarly, a negative regression coefficient indicates a higher probability for the occurrence of tetraploids in the absence of higher‐ploid cytotypes.

The occurrence of neither penta‐, hepta‐, nor octoploids was significantly affected by the occurrence of the other apomictic cytotypes (Table 2).

**TABLE 2:** Effects of the occurrence of the apomictic cytotypes on each other's occurrence in 133 populations derived from single predictor binomial generalized linear regressions

	Coefficients ± *SE*	*z* value	*p* values	*D* ^2^
Pentaploids ↔ Heptaploids	−1.48 ± 0.67	−2.23	0.156	0.06/0.03
Pentaploids ↔ Octoploids	−0.51 ± 0.58	−0.88	1.000	0.00/0.00
Heptaploids ↔ Octoploids	0.35 ± 0.42	0.82	1.000	0.00/0.00

Note

*D* ^2^ is a coefficient of determination (equivalent to *R* ^2^) for generalized linear regression models following Guisan and Zimmermann (2000). For each cytotype combination, all statistics except *D* ^2^ were the same using either cytotype as predictor. Bonferroni corrections were applied to *p* values to adjust for multiple comparisons. The two values of *D* ^2^ refer to the regression of the second cytotype on the first one and vice versa, respectively.

## 4 DISCUSSION

### 4.1 The effect of postglacial colonization history on the current distribution of sexual tetraploid *Potentilla puberula*

During the LGM, the study area was covered with ice except for its southernmost fringe (Ehlers & Gibbard, 2004; Figure 1). Although in situ nunatak survival has been proposed for some alpine and subnival plant and animal species (Escobar García et al., 2012; Pan, Hülber, Willner, & Schneeweiss, 2020; Schönswetter & Schneeweiss, 2019; Stehlik, Blattner, Holderegger, & Bachmann, 2002; Wachter et al., 2016; Westergaard et al., 2019), this option should be reasonably excluded for lowland‐to‐subalpine species like *P. puberula*. Instead, its survival was likely restricted to the area of today's montane forest‐dwelling species, whose hypothetical refugia are situated at the southern and eastern margins of the Eastern European Alps (Heuertz et al., 2004; Magri et al., 2006; Ravazzi, 2002; Slovák, Kučera, Turis, & Zozomová‐Lihová, 2012; Tab erlet, Fumagalli, Wust‐Saucy, & Cosson, 1998; Tribsch & Schönswetter, 2003).

Our molecular data showed that genetic diversity and rarity of sexual tetraploids decline along a southeast–northwest axis. Several molecular studies on Alpine plants support a recolonization of the Alps from southern refugia (e.g., Schönswetter, Stehlik, Holderegger, & Tribsch, 2005). For instance, among montane or subalpine plants, it has been proposed that *Cyclamen purpurascens* has survived in a southern refugium in the area of Lake Garda and in the Karst region in the east (Slovák et al., 2012). A colonization of the Eastern European Alps from the southeast has also been suggested for *Polygonatum verticillatum* (Kramp, Huck, Niketić, Tomović, & Schmitt, 2009) and *Minuartia* series *Laricifoliae* (Moore & Kadereit, 2013). In the case of sexual *P. puberula*, a colonization pathway from southern refugia seems more likely, although populations in East Tyrol exhibited relatively high values of rarity (Figure 2d). This result may favor an east–west colonization route from refugial areas at the eastern margin of the Alps, as has been identified for several alpine species (e.g., Tribsch, Schönswetter, & Stuessy, 2002). However, tetraploid populations of *P. puberula* are rarer in the eastern part of our study area (Figure 1). Their values of rarity may thus reflect isolation or minor phylogeographic differentiation instead (see also Nardi et al., 2018). Moreover, the absence of known tetraploid populations from central and eastern Austria (Dobeš, 1999) further disproves the scenario of a colonization of the surveyed region from an eastern refugium. We therefore propose that tetraploid *P. puberula* colonized the central parts of the Eastern European Alps from glacial refugia at the southern margin of the study area.

The expansion of sexuals into previously glaciated areas is predicted to be hindered by priority effects of apomicts, which are expected to be better and faster colonizers (Baker, 1955; Hörandl, 2009; Stebbins, 1957). Despite this, we detected no effects of colonization history on the current distribution of sexual tetraploid *P. puberula*: that is, we found no significant relation between the cost distances from the southern LGM ice sheet margin and the presence of tetraploids in the studied populations (Table 1; Figure 5). As a thermophilic species hardly reaching the tree line, it likely followed the main valleys during its recolonization of the study area, without encountering substantial migration barriers. Our results refute that apomicts blocked sexuals’ establishment in previously glaciated areas along a latitudinal gradient. Instead, they might have secondarily invaded—or postglacially emerged within—the range of sexuals, possibly from other refugia and promoted by human influence, to which they seem to be associated (Alonso‐Marcos et al., 2019a). Consequently, the current distribution pattern of both sexuals and apomicts is more related to ecological site conditions (i.e., local climate and land use) and particularly reproductive and competitive interaction than to colonization abilities per se.

### 4.2 Occurrence of apomicts is the most important factor of sexuals’ geographic distribution

Our analyses identified the occurrence of apomicts as the most important factor determining the current distribution of sexual *P. puberula*. The presence of each single apomictic cytotype reduced the probability of sexuals to occur within populations (Table 1). Moreover, 31% of the variation in the distribution of sexuals was explained exclusively by the presence or absence of apomictic cytotypes (Figure 5).

The spatial separation and avoidance of sexuals and apomicts can be explained by a variety of reasons, related to either ploidy level or reproductive mode. An increase in ploidy level may determine immediate genome reorganizations or changes in gene expression potentially resulting in plant traits increasing fitness and competitive potential, such as higher vigor and bigger seeds enabling faster seedling growth (Parisod, Holderegger, & Brochmann, 2010; Te Beest et al., 2012). Nucleotypic effects (Levin, 1983), by definition, depend on the number of genome copies and can, hence, be expected to increase with ploidy level. However, we could not find such a relation, as pentaploids, that is, the lowest ploidy level among apomicts, affected sexuals’ occurrence the most, followed by octoploids and heptaploids (Table 1). Moreover, the apomictic cytotypes occurred independently of each other (Table 2). Overall, our results do not provide evidence for a strong effect of ploidy itself on the exclusion of sexuals by apomicts. In contrast, several aspects related to apomixis are likely more crucial. Firstly, female fertility, in terms of seed set, has been shown to be higher in autonomous apomicts (i.e., not requiring pollen for endosperm development) like *Antennaria* (Michaels & Bazzaz, 1986) and *Taraxacum* (van Dijk, 2007) than in their sexual relatives. This could be true for pseudogamous apomicts as well, provided that their pollen is viable (Dobeš, Koch, & Sharbel, 2006; Hörandl, 2008). Secondly, it has been shown that apomixis potentially confers an increased rate of seedling establishment by securing embryo formation and extending its time window beyond anthesis (Espinoza, Pessino, Quarín, & Valle, 2002; Hojsgaard, Schegg, Valls, Martínez, & Quarin, 2008). Finally, apomicts can interfere with sexuals’ reproduction. Apomixis in *P. puberula* is pseudogamous (Müntzing, 1928; Rutishauser, 1969), and pollen is usually viable in apomicts (Alonso‐Marcos, Hülber, Myllynen, Pérez Rodríguez, & Dobeš, 2018; Dobeš et al., 2018). Experimental cross‐fertilizations with pollen from apomicts caused a significant reduction of the seed set of sexual *P. puberula*, as well as an increase of ploidy in some progeny (Alonso‐Marcos et al., 2018; Dobeš et al., 2018). Such a cytological transformation may cause a loss of progeny due to sterility or heritable spread of the apomictic trait (Ozias‐Akins & van Dijk, 2007). In contrast, self‐compatible apomicts do not or only slightly suffer from cross‐pollination by sexuals, resulting in asymmetric reproductive interferences at the expense of sexuals in *P. puberula* (Dobeš et al., 2018). Similar consequences of inter‐cytotype crosses were also observed in experimental crosses conducted in other systems (e.g., Hersh et al., 2016; Nogler, 1984; Quarin, 1999; Savidan, 1980; Tas & Van Dijk, 1999; Záveský, Jarolímová, & Štěpánek, 2007). In most of these cases, one part of the offspring was sterile while another part reproduced by apomixis (reviewed by Ozias‐Akins & van Dijk, 2007). Although the inheritance of apomixis in *P. puberula* is still unknown, the genetic admixture with sexuals found in field‐collected apomictic genotypes (Nardi et al., 2018) proves that recombination between sexuals and apomicts resulting in apomictic offspring rarely occurs in the wild.

Both asymmetric reproductive interference and superior fitness of apomicts could ultimately lead to the displacement of sexuals by apomicts (Joshi & Moody, 1995; Mogie, 1992). Assuming this scenario, the current mosaic‐like distribution of *P. puberula* cytotypes in the Eastern European Alps may be interpreted as a transitional phase inevitably leading to the replacement of sexuals in the study area. However, several processes and plant traits might ensure sexuals’ survival and their coexistence with apomicts. Firstly, negative reproductive interference for sexuals is counterbalanced by a reduction in the frequency of inter‐cytotype fertilization due to prezygotic barriers such as homoploid pollen precedence in tetraploids (Alonso‐Marcos et al., 2018). Secondly, the frequency of inter‐cytotype fertilization is likely to be reduced in case of low pollen quality of apomicts (Britton & Mogie, 2001; Mogie, 2011). In *P. puberula*, pollen quality of apomicts varies strongly among individuals and populations (Alonso‐Marcos et al., 2018; Dobeš et al., 2018). Thirdly, a reduction of sexual progeny may not be demographically relevant if there is a density‐dependent selection during early developmental stages of individuals (Kyogoku, 2015). In that case, only the relative frequency of cytotypes in the population determines the replacement of sexuals (see also Levin, 1975). Finally, limited seed and pollen dispersal result into a cytotype spatial clustering, which strongly contributes to the persistence of sexuals at the local scale (Britton & Mogie, 2001). Such clusters can hardly be penetrated by the opposite reproductive mode, which can be interpreted as blocking at a local scale. This phenomenon can enable long‐term maintenance of the observed mosaic‐like distribution of cytotypes in *P. puberula* (Figure 1).

### 4.3 Joint effects of occurrence of apomicts and ecological preferences on sexuals’ distribution

Because of their relatively easy assessment and relation to geography (Kambach et al., 2019; Slatyer, Hirst, & Sexton, 2013), abiotic requirements generally dominate the research on species distribution (e.g., Pearson & Dawson, 2003). In our analysis, ecological preferences unambiguously explained only 7% of the variation in sexual distribution compatible with the slight ecological differentiation among reproductively differentiated cytotypes (Alonso‐Marcos et al., 2019a). An additional 12% was explained by both ecological preferences and occurrence of apomicts (Figure 5), indicating that the two factors are interrelated. The likely interpretation would be that sexuals tend to occur in cytologically monomorphic populations in their preferred habitats. However, the nature of the interrelation of sexuals’ ecological preferences and apomicts’ occurrence cannot be clearly determined and should be addressed with caution (Borcard, Gillet, & Legendre, 2011). The two factors may simply correlate with no causal relation; that is, ecological differentiation between reproductive modes happened regardless of their competitive interactions, as theoretically could be the case for taxa that evolved independently (i.e., without interacting). However, apomictic *P. puberula* most likely originated from and occasionally backcrossed with sexuals, making this hypothesis implausible (Nardi et al., 2018). Alternatively, ecological differentiation and competitive interactions may be causally linked. On the one hand, reproductive and competitive interactions should drive ecological differentiation, as ecologically diverged genotypes will suffer less from reproductive interference and resource competition (Rushworth, Windham, Keith, & Mitchell‐Olds, 2018; Vrijenhoek, 1984). On the other hand, ecological differentiation can result in a condition of environmentally dependent fitness and interactions between sexuals and apomicts (Gaggiotti, 1994; Rey, Manzaneda, & Alcántara, 2017), provided that ecological habitat preferences are adaptive. Either case suggests a fundamental role of ecological differentiation for the long‐term coexistence of reproductive modes at the geographic scale.

## 5 CONCLUSION

Geographic distributions of taxa result as a consequence of concurring factors. In this study, we applied a variation partitioning analysis to quantify the relative effects that the major factors ecological preferences, colonization history, and occurrence of closely related taxa have on the geographic distribution of sexual‐apomictic taxa. Colonization history of populations and ecological differentiation are hitherto comparatively well‐documented factors governing the geographic distribution of sexuals and apomicts under the influence of cyclic glacial–interglacial climatic change. Here, we demonstrate a prominent role of competitive and reproductive interaction among cytotypes as a modifier of their geographic distribution and suggest that the interactions are interrelated to their observed ecological differentiation. The result thus highlights that competitive and reproductive interactions among sexuals and apomicts can have a high importance for shaping their biogeography as well as their ecological niches.

## CONFLICT OF INTEREST

We have no conflict of interest to declare.

## AUTHOR CONTRIBUTION

**Flavia Domizia Nardi:** Conceptualization (equal); data curation (equal); formal analysis (equal); investigation (equal); methodology (equal); visualization (lead); writing – original draft (lead); writing – review & editing (equal). **Karl Hülber:** Conceptualization (equal); formal analysis (equal); funding acquisition (equal); investigation (equal); methodology (equal); writing – review & editing (equal). **Dietmar Moser:** Data curation (equal); formal analysis (equal); methodology (supporting); visualization (equal); writing – review & editing (supporting). **Henar Alonso‐Marcos:** Conceptualization (supporting); data curation (equal); writing – review & editing (supporting). **Andreas Tribsch:** Conceptualization (equal); funding acquisition (equal); methodology (supporting); supervision (equal); writing – review & editing (supporting). **Christoph Dobeš:** Conceptualization (equal); funding acquisition (lead); investigation (equal); project administration (lead); supervision (equal); writing – review & editing (equal).

## Supporting information

Appendix S1

## Data Availability

Cytotype composition and ecological characterization of the sampled populations are available on Dryad (Alonso‐Marcos et al., 2019b). The presence/absence AFLP matrix and individual cpDNA haplotype assignation were published in Table S5 of Electronic Supplement 2 by Nardi et al. (2018). The cpDNA sequences were published in GenBank (MG995594–MG995691) under the haplotype codes provided in Table S2 of Electronic Supplement 1 by Nardi et al. (2018). The list of populations used within the study, including the calculated cost distance from the LGM border, as well as the chloroplast and nuclear genetic indices is provided as Table S1.
