# Supplementary material for: Occurrence of apomictic conspecifics and ecological preferences rather than colonization history govern the geographic distribution of sexual Potentilla puberula
Source: Ecol Evol. 2020 Jun 22;10(14):7306–19. doi: 10.1002/ece3.6455 (PMC7391561; doi:10.1002/ece3.6455)
Supplement: Supplementary file 1 — Appendix S1 [file ECE3-10-7306-s001.docx]

**Appendix S1**

**Table S1.** General description of 235 sexual, apomictic and mixed sexual-apomictic populations of *Potentilla puberula* sampled in the Eastern European Alps (Alonso-Marcos et al., 2019a, 2019b) including their geographic coordinates and elevation, and cost distance (i.e., elevation-weighted spatial distance) to the southern border of the maximum ice sheet extension at LGM. The chloroplast (cpDNA) and nuclear (AFLP) genetic indices are provided for 68 genotyped tetraploid sexual populations. Of the 238 populations published by Alonso-Marcos et al. (2019a, 2019b), three were excluded either because of geographical isolation (Oberegg) or the composition of solely hexaploid individuals (Bagni Lusnizza and Roveredo).

|  |  |  |  |  |  |  | cpDNA | | AFLP | |
| --- | --- | --- | --- | --- | --- | --- | --- | --- | --- | --- |
| **Population** | **Longitude (°E)** | **Latitude (°N)** | **Country** | **Elevation (m a.s.l.)** | **Reproductive mode** | **Cost distance** | **Nucleotide diversity** | **Haplotype diversity** | **Gene diversity** | **Rarity** |
| Affenhausen | 10.996 | 47.309 | AT | 870 | apomictic | 119887500 |  |  |  |  |
| Ainet | 12.686 | 46.867 | AT | 710 | apomictic | 52586700 |  |  |  |  |
| AlaE | 11.025 | 45.755 | IT | 350 | sexual | 808766 | 0 | 0 | 0.134 | 1.627 |
| Aldein | 11.354 | 46.371 | IT | 1225 | apomictic | 17519930 |  |  |  |  |
| Ambach | 10.866 | 47.220 | AT | 850 | sexual | 109138900 | 0.003 | 0.500 | 0.104 | 1.642 |
| Antholz | 12.099 | 46.847 | IT | 1230 | sexual | 76742820 |  |  |  |  |
| Arco Klettersteig | 10.888 | 45.927 | IT | 270 | sexual | 1384374 |  |  |  |  |
| Arco NE | 10.897 | 45.931 | IT | 110 | sexual | 1412128 | 0.004 | 1 | 0.101 | 0.901 |
| Ardez | 10.204 | 46.773 | CH | 1460 | sexual | 112439500 | 0 | 0.5 | 0.102 | 0.911 |
| Arnig N | 12.632 | 46.984 | AT | 1240 | apomictic | 67974040 |  |  |  |  |
| Arzler Alm | 11.406 | 47.295 | AT | 1000 | apomictic | 106086200 |  |  |  |  |
| Auer Klaunzerberg | 12.559 | 46.975 | AT | 1230 | apomictic | 65861600 |  |  |  |  |
| Avio | 10.930 | 45.734 | IT | 180 | sexual | 398580 | 0.001 | 0.5 | 0.119 | 1.905 |
| Avio 500 | 10.922 | 45.717 | IT | 500 | apomictic | 347977 |  |  |  |  |
| Beseno | 11.105 | 45.930 | IT | 270 | apomictic | 3869686 |  |  |  |  |
| Bichl | 12.539 | 46.985 | AT | 940 | mixed | 67271040 | 0.007 | 1 | 0.129 | 2.445 |
| Birkach | 10.552 | 46.978 | AT | 990 | sexual | 106868900 |  |  |  |  |
| Bobojach | 12.404 | 47.017 | AT | 1380 | mixed | 79739000 |  |  |  |  |
| Boedenalm | 11.698 | 46.923 | IT | 1700 | mixed | 59396400 | 0.004 | 0.833 | 0.11 | 1.181 |
| Bozen Gries | 11.331 | 46.507 | IT | 330 | sexual | 18465890 |  |  |  |  |
| Brenito Belluno | 10.874 | 45.646 | IT | 230 | sexual | 384317 | 0.007 | 0.667 | 0.134 | 1.906 |
| Bruneck | 11.940 | 46.793 | IT | 860 | sexual | 59482150 |  |  |  |  |
| Buchholz | 11.248 | 46.249 | IT | 550 | apomictic | 12335970 |  |  |  |  |
| Burg Berneck | 10.704 | 47.078 | AT | 1120 | sexual | 122737800 |  |  |  |  |
| Burgfrieden | 12.714 | 46.798 | AT | 1100 | apomictic | 50356610 |  |  |  |  |
| Burgstall Kofler | 11.195 | 46.622 | IT | 540 | apomictic | 22568480 |  |  |  |  |
| CampioloW | 13.134 | 46.392 | IT | 300 | mixed | 2535220 | 0.005 | 0.833 | 0.15 | 3.334 |
| Canale | 10.833 | 45.590 | IT | 170 | sexual | 291747 |  |  |  |  |
| Casalicolo | 10.443 | 45.608 | IT | 450 | sexual | 0 |  |  |  |  |
| Castelfeder | 11.291 | 46.336 | IT | 360 | sexual | 14019640 |  |  |  |  |
| Castellano | 11.433 | 46.278 | IT | 950 | sexual | 26329140 | 0.003 | 0.5 | 0.12 | 1.477 |
| Ceraino | 10.828 | 45.574 | IT | 180 | sexual | 147307 |  |  |  |  |
| Dabaklamm | 12.631 | 47.024 | AT | 1490 | apomictic | 74590230 |  |  |  |  |
| Eigenhofen | 11.200 | 47.282 | AT | 640 | mixed | 111325100 |  |  |  |  |
| Elvas | 11.667 | 46.729 | IT | 840 | sexual | 36545080 |  |  |  |  |
| Erlach Forststrae | 12.339 | 46.924 | AT | 1700 | apomictic | 84461140 |  |  |  |  |
| Erlach Gehoeft | 12.339 | 46.922 | AT | 1610 | apomictic | 84253250 |  |  |  |  |
| Erlbach | 12.370 | 46.747 | AT | 1300 | apomictic | 69167310 |  |  |  |  |
| Eyrs | 10.632 | 46.631 | IT | 920 | sexual | 51243150 |  |  |  |  |
| Faggen1 | 10.677 | 47.077 | AT | 920 | sexual | 120567900 |  |  |  |  |
| Faggen2 | 10.675 | 47.078 | AT | 920 | sexual | 120645900 | 0 | 0 | 0.098 | 1.308 |
| Feld SSE | 12.572 | 46.951 | AT | 1000 | mixed | 62907260 |  |  |  |  |
| Feldthurns | 11.600 | 46.664 | IT | 810 | sexual | 31295380 | 0.005 | 0.833 | 0.109 | 1.315 |
| Fineil | 10.826 | 46.742 | IT | 1975 | apomictic | 51232960 |  |  |  |  |
| Finele | 11.165 | 46.699 | IT | 570 | sexual | 25543010 | 0 | 0 | 0.103 | 1.493 |
| Fischleinbach | 12.352 | 46.663 | IT | 1450 | apomictic | 55376130 |  |  |  |  |
| Fiss | 10.625 | 47.051 | AT | 1320 | sexual | 117042100 |  |  |  |  |
| Flans | 11.435 | 46.923 | IT | 1400 | sexual | 58425540 | 0.003 | 0.5 | 0.091 | 1.088 |
| Flathalpe | 10.558 | 47.133 | AT | 1100 | apomictic | 129638500 |  |  |  |  |
| Flie | 10.639 | 47.116 | AT | 950 | sexual | 124868200 |  |  |  |  |
| Flirsch | 10.410 | 47.152 | AT | 1190 | apomictic | 142650200 |  |  |  |  |
| Forte Masua | 10.876 | 45.592 | IT | 960 | sexual | 0 | 0.003 | 0.833 | 0.117 | 1.456 |
| Fosse | 10.912 | 45.641 | IT | 880 | mixed | 0 |  |  |  |  |
| Franzensfeste | 11.630 | 46.777 | IT | 730 | apomictic | 40039770 |  |  |  |  |
| Frosnitztal | 12.503 | 47.045 | AT | 1300 | apomictic | 75674300 |  |  |  |  |
| Gaid | 11.214 | 46.509 | IT | 810 | apomictic | 20149090 |  |  |  |  |
| Gaium Croce | 10.824 | 45.555 | IT | 180 | sexual | 0 | 0.003 | 0.833 | 0.116 | 1.739 |
| Garniga Therme | 11.088 | 46.005 | IT | 820 | apomictic | 5818800 |  |  |  |  |
| Garniga Vecchio | 11.080 | 46.014 | IT | 980 | apomictic | 6629636 |  |  |  |  |
| Gemona | 13.146 | 46.286 | IT | 400 | apomictic | 263250 |  |  |  |  |
| Gonzach | 12.662 | 46.875 | AT | 870 | apomictic | 54263460 |  |  |  |  |
| Graun | 10.545 | 46.808 | IT | 1540 | sexual | 79431960 |  |  |  |  |
| Greit | 10.565 | 46.959 | AT | 1340 | sexual | 107677200 |  |  |  |  |
| Grezzana | 11.010 | 45.535 | IT | 250 | sexual | 0 | 0.004 | 0.667 | 0.106 | 1.548 |
| Grins2 | 10.536 | 47.143 | AT | 910 | apomictic | 130230100 |  |  |  |  |
| Grizzo | 12.644 | 46.158 | IT | 425 | apomictic | 0 |  |  |  |  |
| Groder | 12.332 | 47.018 | AT | 1520 | mixed | 86909680 | 0 | 0 | 0.125 | 1.419 |
| Gruben | 12.514 | 47.048 | AT | 1200 | apomictic | 75265970 |  |  |  |  |
| Grumes | 11.291 | 46.220 | IT | 890 | apomictic | 16356630 |  |  |  |  |
| Guarda | 10.151 | 46.774 | CH | 1600 | sexual | 114219600 |  |  |  |  |
| Guardia | 11.131 | 45.906 | IT | 920 | apomictic | 5583828 |  |  |  |  |
| Hafling Oberdorf | 11.220 | 46.651 | IT | 1340 | apomictic | 25890170 |  |  |  |  |
| Heinfels Schloss | 12.438 | 46.750 | AT | 1120 | mixed | 68696570 | 0 | 0 | 0.11 | 1.69 |
| Hinterbichl N | 12.340 | 47.020 | AT | 1400 | sexual | 86240450 |  |  |  |  |
| Hochmuth | 11.124 | 46.704 | IT | 1450 | sexual | 26543210 | 0 | 0 | 0.112 | 0.962 |
| Hof | 12.497 | 46.926 | AT | 1500 | apomictic | 67909470 |  |  |  |  |
| Hopfgarten | 12.526 | 46.926 | AT | 1250 | apomictic | 64954790 |  |  |  |  |
| Huben | 10.979 | 47.042 | AT | 1250 | apomictic | 85298410 |  |  |  |  |
| Innervillgraten | 12.361 | 46.812 | AT | 1450 | mixed | 82290610 |  |  |  |  |
| Innichen | 12.287 | 46.736 | IT | 1200 | sexual | 69085770 |  |  |  |  |
| Kaltern | 11.282 | 46.385 | IT | 430 | sexual | 15156340 |  |  |  |  |
| Katalalm | 12.491 | 47.057 | AT | 1720 | apomictic | 77918600 |  |  |  |  |
| Kauderle | 11.202 | 46.308 | IT | 600 | apomictic | 13071820 |  |  |  |  |
| Kaunertal Maut | 10.738 | 47.019 | AT | 1375 | apomictic | 122824500 |  |  |  |  |
| Kauns | 10.698 | 47.080 | AT | 1200 | mixed | 122340000 |  |  |  |  |
| Kematen | 11.543 | 46.961 | IT | 1500 | apomictic | 68498900 |  |  |  |  |
| Kiens | 11.852 | 46.799 | IT | 850 | sexual | 53396530 |  |  |  |  |
| Kortsch | 10.764 | 46.635 | IT | 970 | sexual | 42508280 | 0.009 | 0.833 | 0.114 | 1.082 |
| Kosten | 12.602 | 46.786 | AT | 1440 | apomictic | 58929450 |  |  |  |  |
| Ladis | 10.652 | 47.076 | AT | 1150 | sexual | 120274100 | 0 | 0 | 0.083 | 1.707 |
| Ladner | 10.391 | 47.068 | AT | 1240 | apomictic | 133376100 |  |  |  |  |
| Laengenfeld | 10.966 | 47.081 | AT | 1180 | sexual | 91028100 | 0.003 | 0.5 | 0.105 | 1.449 |
| Lafairs | 10.565 | 46.990 | AT | 1100 | sexual | 108649900 | 0 | 0 | 0.099 | 0.856 |
| Lago di Lago | 12.218 | 45.990 | IT | 330 | apomictic | 354537 |  |  |  |  |
| Lana S | 12.632 | 46.986 | AT | 1295 | apomictic | 68228740 |  |  |  |  |
| Lasino | 10.976 | 46.026 | IT | 490 | sexual | 3993767 | 0.002 | 0.833 | 0.114 | 1.72 |
| Latzfons | 11.555 | 46.674 | IT | 1280 | sexual | 32264070 | 0.003 | 1 | 0.124 | 1.622 |
| Lavin | 10.111 | 46.771 | CH | 1460 | sexual | 111414500 |  |  |  |  |
| Luttach | 11.915 | 46.948 | IT | 1070 | mixed | 73202750 | 0 | 0 | 0.098 | 1.086 |
| M Locherboden | 10.963 | 47.282 | AT | 780 | mixed | 116811000 |  |  |  |  |
| Maria Hilf | 12.293 | 46.913 | AT | 1480 | apomictic | 89038840 |  |  |  |  |
| Martell | 10.761 | 46.566 | IT | 1900 | sexual | 47650840 |  |  |  |  |
| Matrei B | 11.450 | 47.123 | AT | 1050 | apomictic | 89465080 |  |  |  |  |
| Matrei NE | 12.544 | 47.008 | AT | 1200 | mixed | 69676130 |  |  |  |  |
| Mattersberger | 12.567 | 46.962 | AT | 1390 | apomictic | 64345300 |  |  |  |  |
| Mauls | 11.523 | 46.855 | IT | 960 | mixed | 50922370 |  |  |  |  |
| Maxer | 12.336 | 46.814 | AT | 1460 | apomictic | 83577580 |  |  |  |  |
| Melag | 10.656 | 46.840 | IT | 1940 | sexual | 93492960 | 0.002 | 0.5 | 0.123 | 1.615 |
| Mellitz | 12.457 | 46.929 | AT | 1400 | apomictic | 71632930 |  |  |  |  |
| Mezzocorona | 11.126 | 46.225 | IT | 840 | mixed | 10512310 |  |  |  |  |
| Mezzomonte | 11.133 | 45.920 | IT | 610 | apomictic | 4794437 |  |  |  |  |
| Mieders | 11.376 | 47.169 | AT | 960 | apomictic | 99901860 |  |  |  |  |
| Mignano | 11.707 | 45.828 | IT | 200 | mixed | 0 | 0 | 0 | 0.102 | 0.867 |
| Monreale | 11.156 | 46.207 | IT | 460 | sexual | 10133250 | 0 | 0 | 0.127 | 1.473 |
| Moos Passeier | 11.168 | 46.832 | IT | 1050 | apomictic | 38023300 |  |  |  |  |
| Mosson | 11.440 | 45.787 | IT | 500 | mixed | 0 |  |  |  |  |
| Muehlbach | 11.977 | 46.848 | IT | 1540 | sexual | 64084320 | 0 | 0.5 | 0.089 | 0.821 |
| Nals | 11.186 | 46.530 | IT | 950 | apomictic | 21234090 |  |  |  |  |
| Nals Wehrburg | 11.188 | 46.550 | IT | 560 | mixed | 20897790 |  |  |  |  |
| Nauders | 10.510 | 46.894 | AT | 1500 | sexual | 94374780 | 0 | 0 | 0.116 | 1.294 |
| Niedermauern | 12.434 | 47.002 | AT | 1220 | sexual | 76148390 |  |  |  |  |
| Oberassling | 12.638 | 46.789 | AT | 1220 | apomictic | 55817650 |  |  |  |  |
| Oberbergtal | 11.262 | 47.118 | AT | 1350 | apomictic | 95717500 |  |  |  |  |
| Oberbichl | 12.362 | 47.022 | AT | 1540 | sexual | 84393250 |  |  |  |  |
| Oberdrauburg | 12.964 | 46.754 | AT | 730 | apomictic | 35792140 |  |  |  |  |
| Obergaimberg | 12.782 | 46.846 | AT | 1060 | apomictic | 47188920 |  |  |  |  |
| Obermauern | 12.435 | 47.005 | AT | 1300 | sexual | 76233840 | 0 | 0 | 0.139 | 2.139 |
| Oberpeischlach | 12.594 | 46.936 | AT | 1100 | apomictic | 60782070 |  |  |  |  |
| Obersteiner | 12.409 | 47.018 | AT | 1460 | mixed | 79749570 | 0.003 | 0.5 | 0.147 | 2.117 |
| Olang | 12.043 | 46.771 | IT | 1010 | mixed | 67609380 |  |  |  |  |
| Ossenigo | 10.909 | 45.674 | IT | 250 | mixed | 405715 | 0.004 | 0.8 | 0.112 | 1.298 |
| Passeier Spath | 11.207 | 46.762 | IT | 650 | apomictic | 29002410 |  |  |  |  |
| Passeier-Moos | 11.196 | 46.823 | IT | 890 | apomictic | 35580050 |  |  |  |  |
| Patsch | 11.405 | 47.210 | AT | 960 | apomictic | 99295930 |  |  |  |  |
| Pavone | 10.438 | 45.654 | IT | 330 | sexual | 0 | 0.003 | 0.833 | 0.114 | 1.315 |
| Perdross | 10.578 | 46.814 | IT | 1700 | mixed | 83122660 | 0.001 | 0.667 | 0.114 | 1.252 |
| Pettnau | 11.125 | 47.306 | AT | 690 | sexual | 115388000 | 0.006 | 1 | 0.12 | 2.3 |
| Pflersch Ende | 11.330 | 46.970 | IT | 1450 | apomictic | 65350340 |  |  |  |  |
| Pfossertal Abzw | 10.918 | 46.706 | IT | 1250 | sexual | 38673760 | 0 | 0 | 0.122 | 1.527 |
| Pfunders | 11.698 | 46.904 | IT | 1460 | sexual | 55756850 |  |  |  |  |
| Pians | 10.506 | 47.132 | AT | 910 | apomictic | 132449100 |  |  |  |  |
| Planeil | 10.577 | 46.722 | IT | 1645 | sexual | 66446500 |  |  |  |  |
| Plenten | 11.536 | 47.046 | AT | 1350 | apomictic | 80675150 |  |  |  |  |
| Pontebba | 13.313 | 46.510 | IT | 600 | apomictic | 13139600 |  |  |  |  |
| Praegraten N | 12.375 | 47.021 | AT | 1400 | sexual | 82871700 | 0 | 0 | 0.106 | 2.353 |
| Praemajur | 10.510 | 46.704 | IT | 1720 | sexual | 63788420 |  |  |  |  |
| Prags | 12.136 | 46.725 | IT | 1220 | sexual | 77814270 |  |  |  |  |
| Punt de la Resgia | 10.410 | 46.835 | CH | 1095 | sexual | 97597870 |  |  |  |  |
| Raas W | 11.653 | 46.747 | IT | 750 | sexual | 37404970 | 0.006 | 0.833 | 0.101 | 1.151 |
| Rabenstein | 12.466 | 47.009 | AT | 1380 | apomictic | 74067540 |  |  |  |  |
| Ramosch | 10.374 | 46.837 | CH | 1430 | sexual | 98821250 |  |  |  |  |
| Raneburg | 12.528 | 47.067 | AT | 1270 | apomictic | 78342940 |  |  |  |  |
| Ratzell | 12.539 | 46.925 | AT | 1190 | apomictic | 63783630 |  |  |  |  |
| Raut | 12.575 | 46.781 | AT | 1450 | mixed | 60823500 | 0.004 | 0.667 | 0.096 | 1.136 |
| Reifenstein | 11.443 | 46.879 | IT | 960 | sexual | 55976410 | 0 | 0 | 0.087 | 0.848 |
| Ridnaun | 11.309 | 46.915 | IT | 1390 | sexual | 53515550 | 0.006 | 0.667 | 0.109 | 0.799 |
| Rodenegg | 11.691 | 46.775 | IT | 880 | sexual | 40639630 |  |  |  |  |
| Ronchi | 11.070 | 45.742 | IT | 800 | apomictic | 986202 |  |  |  |  |
| Roppen | 10.817 | 47.221 | AT | 750 | mixed | 111472100 |  |  |  |  |
| Rossbach | 10.872 | 47.315 | AT | 1000 | apomictic | 120740000 |  |  |  |  |
| S. Valentino | 10.908 | 45.778 | IT | 1270 | apomictic | 0 |  |  |  |  |
| Saeben Burg | 11.570 | 46.646 | IT | 670 | sexual | 29542830 |  |  |  |  |
| Saeben N | 11.581 | 46.652 | IT | 730 | mixed | 30091830 | 0.005 | 1 | 0.1 | 1.01 |
| San Eusebio | 10.363 | 45.611 | IT | 630 | sexual | 0 | 0 | 0 | 0.114 | 1.879 |
| Santantonio | 10.938 | 45.858 | IT | 360 | sexual | 759089 | 0.006 | 0.667 | 0.104 | 1.067 |
| Santosso | 11.382 | 45.742 | IT | 400 | sexual | 0 | 0.005 | 0.667 | 0.113 | 1.503 |
| Scaiola | 10.364 | 45.534 | IT | 200 | mixed | 0 | 0.006 | 1 | 0.104 | 1.715 |
| Schlaneid | 11.234 | 46.579 | IT | 1160 | sexual | 21699100 |  |  |  |  |
| Schluderns | 10.585 | 46.668 | IT | 1010 | sexual | 56983060 | 0.001 | 0.5 | 0.116 | 1.25 |
| Schmirn | 11.545 | 47.073 | AT | 1400 | apomictic | 85555240 |  |  |  |  |
| Schmuders | 11.462 | 46.914 | IT | 1430 | sexual | 59169870 |  |  |  |  |
| Schnalstal | 10.880 | 46.724 | IT | 2060 | sexual | 43605180 |  |  |  |  |
| Schnann | 10.380 | 47.154 | AT | 1210 | apomictic | 144875900 |  |  |  |  |
| Schrottendorf | 12.674 | 46.792 | AT | 1180 | apomictic | 53202000 |  |  |  |  |
| Scuol-Ftan | 10.266 | 46.791 | CH | 1400 | sexual | 105443000 |  |  |  |  |
| Sent | 10.350 | 46.817 | CH | 1330 | sexual | 97451960 | 0.006 | 1 | 0.092 | 0.986 |
| Serravalle | 11.014 | 45.846 | IT | 220 | mixed | 1814074 |  |  |  |  |
| Sigmundskron | 11.305 | 46.480 | IT | 340 | sexual | 17683460 | 0 | 0 | 0.114 | 1.829 |
| Silz N | 10.919 | 47.272 | AT | 690 | sexual | 114391700 | 0 | 0 | 0.113 | 1.398 |
| Sinsen | 10.354 | 47.054 | AT | 1290 | apomictic | 133384100 |  |  |  |  |
| Soelden | 11.013 | 46.960 | AT | 1400 | apomictic | 72213060 |  |  |  |  |
| Soell | 11.243 | 46.355 | IT | 360 | sexual | 14129910 | 0 | 0 | 0.127 | 1.922 |
| Soelles | 10.563 | 46.654 | IT | 930 | sexual | 56842700 |  |  |  |  |
| Sonneburg | 11.894 | 46.789 | IT | 860 | sexual | 56331420 | 0.006 | 1 | 0.087 | 1.109 |
| Sprechenstein | 11.456 | 46.884 | IT | 1070 | sexual | 56283790 |  |  |  |  |
| St. Catarina | 13.401 | 46.503 | IT | 690 | apomictic | 14847030 |  |  |  |  |
| St. Jakob | 11.991 | 47.001 | IT | 1110 | mixed | 81487790 | 0.006 | 0.667 | 0.134 | 1.348 |
| St. Justina | 12.585 | 46.789 | AT | 1200 | mixed | 60258500 |  |  |  |  |
| St. Magdalena | 12.237 | 46.837 | IT | 1470 | apomictic | 90124300 |  |  |  |  |
| St. Peter | 12.059 | 47.023 | IT | 1350 | sexual | 88914660 | 0.006 | 0.667 | 0.104 | 1.077 |
| St. Sigmund | 11.786 | 46.819 | IT | 1160 | mixed | 49681360 | 0 | 0 | 0.095 | 1.253 |
| St. Sigmund Sellrain | 11.113 | 47.204 | AT | 1550 | apomictic | 122510700 |  |  |  |  |
| St. Ulrich | 11.635 | 46.584 | IT | 1130 | apomictic | 32873930 |  |  |  |  |
| St. Veit | 12.422 | 46.927 | AT | 1480 | apomictic | 75166220 |  |  |  |  |
| Staben | 10.964 | 46.648 | IT | 650 | sexual | 31789940 |  |  |  |  |
| Stein | 12.528 | 47.026 | AT | 1300 | apomictic | 72059510 |  |  |  |  |
| Stein S | 10.580 | 46.997 | AT | 960 | sexual | 109722800 |  |  |  |  |
| Stroeden | 12.318 | 47.018 | AT | 1400 | apomictic | 88431500 |  |  |  |  |
| Strumerhof | 12.518 | 47.010 | AT | 1450 | apomictic | 70948600 |  |  |  |  |
| Tappein | 11.160 | 46.679 | IT | 380 | sexual | 24476940 |  |  |  |  |
| Taufers | 11.949 | 46.923 | IT | 940 | sexual | 69400140 |  |  |  |  |
| Terlan Mendel | 11.242 | 46.431 | IT | 600 | sexual | 16690510 | 0.006 | 1 | 0.114 | 1.401 |
| Terlan N | 11.254 | 46.544 | IT | 710 | sexual | 19997680 |  |  |  |  |
| Thaur | 11.487 | 47.295 | AT | 620 | sexual | 107539000 | 0.003 | 0.667 | 0.111 | 1.155 |
| Thurn Ruine | 12.114 | 46.763 | IT | 1180 | sexual | 74012200 | 0.001 | 0.5 | 0.119 | 1.395 |
| Tisens | 11.548 | 46.570 | IT | 920 | apomictic | 26069360 |  |  |  |  |
| Toblach | 12.214 | 46.743 | IT | 1330 | sexual | 74889190 | 0.004 | 1 | 0.104 | 1.069 |
| Toldern | 11.577 | 47.092 | AT | 1600 | apomictic | 90255340 |  |  |  |  |
| Trostburg | 11.536 | 46.585 | IT | 1010 | mixed | 25982460 | 0.005 | 0.5 | 0.108 | 1.053 |
| Tschoetscher H | 11.646 | 46.705 | IT | 680 | sexual | 34681820 | 0.002 | 0.5 | 0.12 | 2.415 |
| Unteralbkus | 12.692 | 46.875 | AT | 1225 | apomictic | 53366860 |  |  |  |  |
| Unterfederaun | 13.812 | 46.570 | AT | 540 | apomictic | 9430446 |  |  |  |  |
| Unterfennberg | 11.180 | 46.273 | IT | 1050 | apomictic | 13167910 |  |  |  |  |
| Unterleibning | 12.636 | 46.903 | AT | 810 | apomictic | 56719900 |  |  |  |  |
| Unterparggen | 12.364 | 46.747 | IT | 1410 | apomictic | 69114020 |  |  |  |  |
| Unterwaldalm | 12.536 | 47.077 | AT | 1450 | apomictic | 80027450 |  |  |  |  |
| Uttenheim | 11.937 | 46.869 | IT | 1160 | sexual | 64516490 |  |  |  |  |
| Vellau Leiteralm | 11.111 | 46.706 | IT | 1570 | sexual | 27243550 |  |  |  |  |
| Vellau W | 11.103 | 46.693 | IT | 910 | sexual | 25895990 |  |  |  |  |
| Vergein | 12.584 | 46.794 | AT | 1382 | apomictic | 60926440 |  |  |  |  |
| Vernagt | 10.851 | 46.739 | IT | 1760 | mixed | 48413040 | 0 | 0 | 0.104 | 1.378 |
| Vill | 11.406 | 47.237 | AT | 850 | sexual | 101875900 | 0.001 | 1 | 0.073 | 1.799 |
| Vinaders | 11.472 | 47.033 | AT | 1210 | apomictic | 77592920 |  |  |  |  |
| Virgen | 12.459 | 47.006 | AT | 1240 | apomictic | 74426010 |  |  |  |  |
| Voels N | 11.498 | 46.526 | IT | 800 | apomictic | 22484200 |  |  |  |  |
| Wassermann | 11.829 | 46.894 | IT | 1200 | sexual | 64869740 |  |  |  |  |
| Welschnofen | 11.544 | 46.433 | IT | 1200 | apomictic | 29902800 |  |  |  |  |
| Zabernig | 12.517 | 47.006 | AT | 1340 | apomictic | 70288980 |  |  |  |  |
| Zaunhof | 10.822 | 47.100 | AT | 1350 | sexual | 112120100 |  |  |  |  |
| Zedlach NW | 12.490 | 47.004 | AT | 1040 | apomictic | 71875510 |  |  |  |  |
| Zernez | 10.102 | 46.698 | CH | 1550 | sexual | 97811440 |  |  |  |  |
| Zerzertal | 10.517 | 46.750 | IT | 1810 | mixed | 70680250 |  |  |  |  |
| Zirl Ruine | 11.239 | 47.278 | AT | 720 | sexual | 109504900 | 0.001 | 0.5 | 0.118 | 1.864 |
| Zoesen | 11.775 | 46.919 | IT | 1720 | sexual | 63279740 | 0.011 | 1 | 0.136 | 1.569 |
| Zwischenwasser | 11.894 | 46.723 | IT | 1160 | mixed | 58799000 |  |  |  |  |
